# Representations of Ultra-Processed Foods: A Global Analysis of How Dietary Guidelines Refer to Levels of Food Processing

**DOI:** 10.34172/ijhpm.2022.6443

**Published:** 2022-02-16

**Authors:** Daniela Koios, Priscila Machado, Jennifer Lacy-Nichols

**Affiliations:** ^1^Melbourne School of Population and Global Health, University of Melbourne, Melbourne, VIC, Australia.; ^2^Institute for Physical Activity and Nutrition (IPAN), School of Exercise and Nutrition Sciences, Deakin University, Melbourne, VIC, Australia.; ^3^Centre for Health Policy, Melbourne School of Population and Global Health, University of Melbourne, Melbourne, VIC, Australia.

**Keywords:** Dietary Recommendations, Ultra-Processed Food, Nutritionism, Food-Based, Nutrition Policy

## Abstract

**Background:** As evidence grows about negative health impacts of ultra-processed foods (UPFs), nutrient-centred advice is contested, and food-based dietary guidelines are increasingly utilised. Previous analyses of dietary guidelines evaluated their potential impact on health and sustainability, but little research has been conducted to examine how the concept of UPFs is reflected in dietary advice for consumers. This paper systematically analyses whether and how UPFs are represented in dietary guidelines internationally.

**Methods:** Based on a systematic online search, the consumer-targeted key messages of 106 dietary guidelines were identified and a qualitative content analysis was conducted. A coding framework was developed to classify messages as ‘eat more’ or ‘eat less’ according to the language used (eg, ‘choose’ vs ‘avoid’) and to differentiate between a focus on nutrients or food processing. Specific foods mentioned in ‘eat less’ guidelines were classified according to their level of processing using the NOVA framework.

**Results:** 99% of guidelines utilised some type of nutrient-based message, either promoting ‘positive’ nutrients (eg, vitamins) or discouraging the consumption of ‘negative’ nutrients (mainly salt, sugar and fat). Explicit references to food processing were present in 45% of ‘eat less’ guidelines and 5% of ‘eat more’ guidelines. Implicit references (eg, promoting ‘raw’ or discouraging ‘packaged’ foods) were found in 43% of ‘eat less’ and 75% of ‘eat more’ guidelines. 53% of the specific foods referred to in ‘eat less’ advice were UPFs.

**Conclusion:** Overall, nutrient-based messages were more common than messages about processing levels. The majority of discouraged foods were UPFs, however some minimally processed foods were discouraged, which points to tensions and contradictions between nutrient- and processing-based dietary advice. As dietary guidelines begin to include advice about food processing, it is important to consider both consumer understanding of the terms used and their capacity to act on the advice.

## Background

**Key Messages**

** Implications for policy makers**

- Dietary guidelines can incorporate advice about ultra-processed foods (UPFs) in three ways: (1) by specifically referring to ‘ultra-processing’ or ‘ultra-processed foods,’ (2) by discouraging specific (ultra-processed) foods, or (3) by referring to characteristics that imply the presence or absence of ultra-processing.
- Food-based advice can reinforce or confuse messages about ultra-processing; to avoid confusion, policy makers can ensure that ‘eat less’ messages are focused on UPFs.
- While some dietary guidelines include messages about processed or UPFs, guidelines could be strengthened by explaining the different levels of processing more clearly.
- To ensure that messages about UPFs are understood and interpreted by consumers, policy-makers can use the terms identified in this study to inform first drafts of dietary guidelines to ensure local and culturally specific suitability of nutrition advice.

** Implications for the public**

While evidence about the harms of ultra-processed foods (UPFs) is growing, dietary advice for the public does not incorporate these findings consistently. On a global scale, dietary messages are often based on nutrients rather than food processing levels. When processing was discussed, guidelines favoured implicit or euphemistic terms, which do not consistently differentiate between levels of processing, and could confuse consumers. Similarly, while most discouraged foods were ultra-processed, some were minimally processed, highlighting inconsistencies in how advice about the harms of UPFs are communicated. This could present challenges for public understanding or implementation of dietary advice. Future dietary guidelines would benefit from the development of actionable and consumer-friendly messages about UPFs.

Over the past decade, nutrition research and dietary advice have increasingly focused on levels of food processing, specifically the health harms associated with the consumption of ultra-processed foods (UPFs).^1,2^ In 2010, the NOVA framework was published as a food classification system to distinguish foods based on their industrial processing levels, ranging from ‘unprocessed’ to ‘ultra-processed.’^3^ The focus on UPFs is not without contestation.^4-6^ Indeed, food processing per se is not a problem as most foods consumed by humans require some form of processing (eg, cleaning, cooking). However, UPFs are distinguished by the industrial *nature *and *purpose* of their processing – often to create foods with novel physical and chemical compositions (eg, by using modified substances such as protein isolates, food colouring or emulsifiers to imitate sensory properties of fresh foods). Few national nutrition policies are based on levels of food processing. However, a small number of dietary guidelines use the NOVA framework as a basis to evaluate quality and harms of foods and diets based on processing levels.^7,8^

 As evidence mounts about the harms of UPFs, it is important to understand how this is translated into nutrition policy in practice. Presently, little is known about whether or how the concept of food processing is incorporated into dietary guidelines around the world. This paper explores how advice about UPFs is incorporated into consumer-directed dietary advice and presents the first systematic analysis of national dietary guidelines in this context. This study analyses two dimensions of dietary advice: (1) the language and terms used to refer to UPFs and food processing more generally, and (2) the specific foods mentioned in dietary advice to evaluate the extent to which ‘foods to limit’ are or are not ultra-processed.

In the early 20th century, food scarcity and nutrient deficiencies were the prevalent health issues of that time and dietary advice therefore aimed to address these deficiencies by promoting the consumption of certain nutrient-rich foods.^9,10^ Subsequently, ‘food groups’ such as fruit and vegetables or protein-rich foods were defined, mostly recommending to eat more of certain foods based on their nutrient-content.^11^ This involved so-called recommended daily allowances focussed on overall energy intake and certain ‘positive’ or ‘protective’ nutrients, such as iron, calcium and vitamins, which suited the dominant nutrition issues of that period (predominantly nutrient deficiencies).^9,12^

From the 1950s onwards, dietary patterns in industrialised nations changed fundamentally as the food supply improved through advances in farming, manufacturing and the rise of supermarkets.^13^ As nutrient deficiencies became less common, the focus shifted to the relationship between diets and NCDs, emphasising how consumption of ‘negative’ nutrients can lead to NCDs.^9,12^ Excessive food consumption was soon identified as an important health issue, and the language of dietary guidelines shifted from a message of “eat more” to “eat less,”^11^ or other “negative nutritional messages.”^12^ An “overly reductive focus”^14^ on nutrients to understand and evaluate the healthfulness of foods or diets has underpinned dietary research and advice for decades, often termed ‘nutritionism.’^11,12,15^ More recently, this reductive approach to health and nutrition has faced growing criticism, as nutrition science has evolved to show that foods and diets are more than the sum of nutrients they contain.^16,17^ Over the last decade, some researchers have shifted away from a nutrient-centric approach to assess the quality and harms of foods based on food processing.^18^

To distinguish foods based on processing characteristics rather than nutrients exclusively, Monteiro et al^3^ developed the NOVA food classification system. The NOVA framework offers a classification system that goes beyond the reductive nutrient-focus by assessing foods according to the extent and purpose of industrial processing they undergo. It provides one of the most comprehensive ways to differentiate levels of industrial food processing^19^ and is widely used for food classification in health research.^20,21^ This framework incorporates four levels of processing; examples and a description of each group can be found in Table 1.

**Table 1:** NOVA Food Classification System^a^

**NOVA**	**Description**
Group 1: Unprocessed or minimally processed foods	Unprocessed: edible parts of plants or of animals/minimally processed: unprocessed foods altered by minimal industrial processes (eg, drying, grinding, roasting, pasteurisation, refrigeration, freezing, vacuum packaging). For example: Fresh, squeezed, chilled, frozen, or dried fruits and leafy and root vegetables; grains such as brown or white rice; legumes; starchy roots and tubers; fungi; meat, poultry, fish and seafood; eggs; fresh or pasteurized milk; fresh or pasteurised fruit or vegetable juices (with no added sugar, sweeteners or flavours); flakes or flour made from corn, wheat, oats or other grains.
Group 2: Processed culinary ingredients	Substances obtained directly from group 1 foods or from nature by industrial processes (eg, centrifuging, refining, extracting or mining), used to prepare group 1 foods. For example: Vegetable oils; butter and lard; sugar; honey; starches extracted from corn and other plants, salt.
Group 3: Processed foods	Manufactured products made by adding salt, oil, sugar or other group 2 ingredients to group 1 foods, using industrial preservation methods such as canning and bottling, non-alcoholic fermentation etc. For example: Canned or bottled fruit, vegetables and legumes; salted, dried, cured, or smoked meats and fish; canned fish (with or without added preservatives); freshly made unpackaged breads and cheeses.
Group 4: UPFs	Formulations of ingredients, mostly of exclusive industrial use, that result from a series of industrial processes (eg, fractioning of whole foods into substances, chemical modifications, using industrial techniques such as hydrolysis, hydrogenation, extrusion, moulding and pre-frying. Ingredients often include sugar, oils and fats, and salt, and substances of no or rare culinary use such as high fructose corn syrup, hydrogenated oils, protein isolates, and ‘cosmetic’ additives such as flavours, flavour enhancers, colours, emulsifiers). For example: soft drinks; sweet or savoury packaged snacks; confectionery; ice-cream; mass-produced packaged breads; margarines; cakes, and cake mixes; breakfast ‘cereals,’ ‘fruit’ yoghurts; infant formulas, many ready to heat products, reconstituted meat products, powdered and packaged ‘instant’ sauces/soups/noodles/desserts.

Abbreviation: UPFs, Ultra-processed foods. ^a^Adapted from Monteiro et al.^3^

This framework recognises that the nature, extent, and purpose of food processing differ widely. For example, processes to make plants or animal-derived foods edible and less perishable have always been part of human cultures. In many ways, food processing has contributed to the development of modern societies, for instance by substantially decreasing the time spent on food preparation and through dramatically increased food safety and security.^22,23^ In contrast, the purpose of cosmetic additives found in many UPFs is to imitate sensorial properties of whole foods, disguise unpleasant flavours that come with processing, or to make foods more attractive to taste, smell, feel and see. This way, UPFs can be overconsumed and easily replace less processed foods. For example, foods with additives “to protect original properties or prevent proliferation of microorganisms”^24^ are not automatically considered UPFs and can be consumed regularly, whereas foods containing substances to create ‘hyper-palatability’ (eg, emulsifiers or flavours) are UPFs and should not be recommended as part of a healthy diet.^24^ Thus, the purpose of processing is a crucial point of differentiation.

Today, the yearly amount of UPFs consumed has reached over 100 kg per capita in most high-income countries and UPF-sales have been steadily increasing in low- and middle-income countries, presenting a growing challenge for public health authorities around the world.^7^ While the amount of UPFs consumed varies substantially between countries and regions, UPFs have become omnipresent in the global food landscape,^7^ increasingly distorting traditional diets around the world.^25,26^ In recent years, evidence has been growing that UPF consumption affects human health negatively.^1,18^ The major health concerns in this context are obesity and related NCDs such as diabetes, hypertension, cardiovascular diseases, and cancer.^1,20,21,27-30^ While causality is difficult to establish in this context, plausible explanations lie in the novel physical structures and/or chemical compositions of the foods (eg, deconstruction of the food matrix, their high salt or sugar content, presence of advanced glycation end products, additives or contact and packaging materials with pro-inflammatory and metabolic disturbances potential),^1,31^ but aggressive marketing strategies that stimulate overconsumption of these foods may also play an important role.^7^ Furthermore, the use of low-cost ingredients like high-fructose corn syrup or hydrogenated vegetable fat combined with ‘cosmetic’ additives are can create so-called ‘hyper-palatability,’ ie, texture and taste that increase the desire to eat more of these products, which amplifies related health issues further.^34,35^ The extent to which food is processed varies greatly and many of today’s industrially processed products have “little if any direct relation to whole foods.”^12^

The rise of obesity and non-communicable diseases (NCDs) represent a global public health challenge and nutrition plays a central role.^36,37^ One policy instrument in this context is the provision of dietary guidelines, which have been utilised for decades by public health and nutrition authorities to educate consumers about healthy eating and to inform other policy approaches, such as food procurement policies.^9,38,39^ Generally, food-based advice is considered more consumer-friendly than nutrient-centred advice.^40^ Consequently, food-based dietary guides have been increasingly utilised over the last decades^9,12,41,42^ However, while food-based terminology is almost universally used for ‘eat more’ advice, Baker et al found that most ‘eat less’ messages in food-based dietary guides still “adopt a nutrient-centric approach, recommending to limit foods high in certain risk nutrients or ‘energy-dense and nutrient-poor’ foods.”^7^

In the seminal book *Food Politics*^11^, Nestle analysed how the food industry opposed dietary advice to eat less of particular foods, and instead lobbied for more positive language to “choose” diets low in saturated fat or to “moderate” consumption. This also included the use of nutritional “euphemisms”^11^ in place of specific food groups, such as providing advice to reduce consumption of foods high in saturated fats, as opposed to meat or dairy products. Advice about which foods to ‘choose’ was often justified by the need to reduce fat-, sugar- or salt-intake.^11,12^ This had the net impact of softening negative messages about particular foods or food groups towards certain ‘nutrients to limit,’ a trend that persists today.^43^ As dietary guidelines begin to incorporate advice about UPFs, it is important to consider the language used to discuss food processing and whether dietary guidelines also use euphemisms to refer to UPFs.

Previous research analysing dietary guidelines has assessed the healthiness and sustainability of the dietary patterns recommended,^8,45,46^ including comparative analyses within certain regions.^47,48^ Studies have also compared the representation of specific food groups (eg, vegetables) and nutrients (eg, fat) in national dietary guidelines.^8,49^ Despite the mounting evidence regarding increased disease risks associated with consumption of UPFs, little is known about how dietary guidelines refer to the concept of ultra-processing or health concerns about food processing more generally.

 This study addresses this research gap by conducting a global analysis of consumer-targeted key messages in dietary guidelines to assess how the concept of UPFs is reflected in ‘eat more’ and ‘eat less’ messages. It was assessed how common the use of nutrient-centric language was, and whether specific terms like “(ultra-)processed” or alternative terms were used to imply that foods are processed or unprocessed. Furthermore, specific foods mentioned in ‘eat less’ messages were categorised according to the NOVA system. This study offers the first systematic analysis of whether and how UPFs and levels of processing are referred to consumer-directed dietary guidelines.

## Methods

A systematic online search was conducted in July 2020 to identify existing dietary guidelines. The World Bank^50^ list of economies was used to generate an initial list of 218 countries which could have dietary guidelines. The website of the Food and Agriculture Organization (FAO)^51^ provided summaries of dietary guidelines from 94 countries including links to the original sources. A basic search of national government websites in English, German^[1]^ or French identified further guidelines. Overall, 137 countries were identified to either have their own dietary guideline (119 countries) or share one with other countries (18 countries). While documents in French were included in the total number of existing guidelines, they could not be analysed due to language restrictions. After excluding non-English and non-German documents, as well as those for specific target groups (eg, children), 106 dietary guidelines were identified and screened for consumer-targeted key messages. Dietary guidelines were available in three formats:

1. Short guide (15 in total: defined as maximum of two pages, eg, a leaflet or brochure with key messages, primarily directed at consumers),
2. Detailed guide (39 in total: written document with detailed information, sometimes for specific target groups such as health professionals),
3. FAO summary (52 in total: high-level translation of key messages; used where no English or German original was available).

Where available, short guides were chosen for analysis, and detailed guidelines were preferred over FAO summaries. Where detailed guidelines were used, key messages (eg, ‘10 steps to healthy diets’/Brazil) were assessed; subsections were only assessed to the extent of which one would assume that a consumer would approach the content (eg, highlighted sections or text boxes). Details about which sections were assessed for each guide can be found in found in Supplementary file 1.

**Figure 1:**
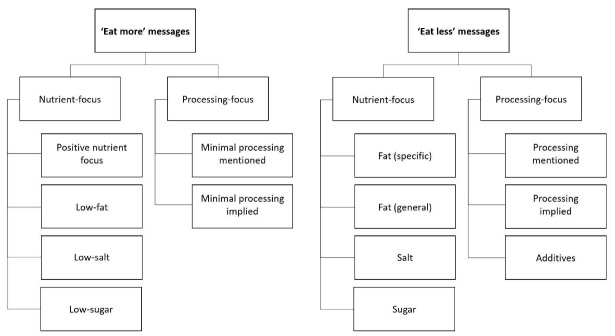
Phase 1 Coding Framework.

Two phases of analysis were undertaken. First, key messages were analysed to identify whether and how they provided advice about nutrients or processing. A representative sample of guidelines was used to develop a coding framework. Nestle’s^11^ distinction between ‘eat more’ guidelines (signified by positive language encouraging consumption such as ‘choose’ or ‘enjoy’) and ‘eat less’ guidelines (signified by negative language discouraging consumption such as ‘avoid’ or ‘limit’) was used to classify messages into two first-level categories. Messages were then segmented into nutrient-focused or processing-focused messages, and data-driven sub-categories were used to further segment the messages (see Figure 1). Nutrient-focused messages were grouped according to the specific nutrient mentioned, and processing-focused messages were classified according to whether they referred to processing explicitly (eg, using the terms ‘unprocessed,’ ‘highly processed’ or ‘ultra-processed) or implicitly (eg, using terms like ‘raw,’ ‘fresh,’ ‘whole’ to imply a lack of processing or terms like ‘packaged,’ ‘fast food’ or ‘junk food’ to imply a high level of processing). References to additives were also commonly associated with food processing messages. Detailed definitions with rules and examples for each category can be found in in Supplementary file 2.

 In the first phase of analysis, the aim was not to quantify how often each country refers to nutrients or processing in its messages, but rather to assess whether or not a country used nutrient-focused or processing-focused language in its consumer-directed messages. Consequently, only one statement per sub-subcategory in each country was recorded (702 quotes in total). Where there were multiple examples of the same sub-subcategory only one illustrative quote was documented (based on which appeared first in the original text). The results indicate whether a country made at least one reference to each sub-subcategory and provide an initial snapshot of which countries have incorporated language about processing into their dietary advice.

In the second phase, specific foods mentioned in ‘eat less’ advice were classified according to the NOVA system. Positive food-based messages (eg, ‘eat more vegetables’) were analysed in previous research^8,49^ and were therefore not systematically assessed; however, they were noticed to be present in all guidelines. Phase 2 aimed to quantify which foods were mentioned most frequently across dietary guidelines (as opposed to within). Therefore, if a guideline referred to a specific food more than once, only one occurrence was counted per guideline. Foods were classified into the four NOVA-food groups (1-4) and one additional group (0) for terms that lacked sufficient information to be classified (Supplementary file 3).

To facilitate comparisons across food groups, foods were then categorised in thirteen overarching food groups, derived from the FAO’s^52^ food groups: Cereals and their products; Pulses, seeds and nuts and their products; Milk and milk products; Meat, eggs and their products; Fish, shellfish and their products; Vegetables and their products; Fruits and their products; Fats and oils; Sweets and sugars; Spices and condiments; Beverages; Composite dishes; and Savoury snacks. Supplementary file 3 details which FAO food groups were used for coding and how foods were classified.

## Results

###  Nutrient vs. Processing-Focus in Dietary Guidelines 

**Figure 2:**
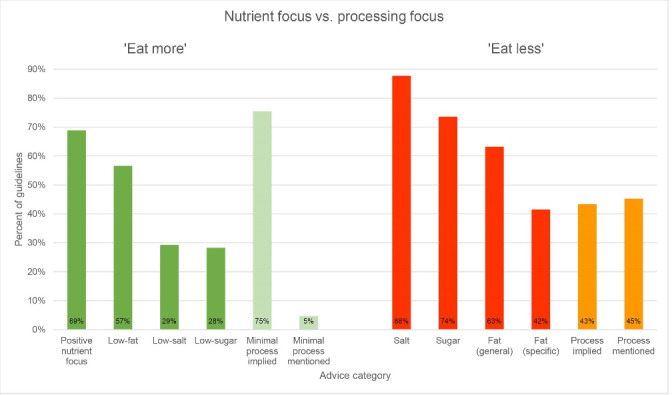
Nutrient-Focus vs. Processing-Focus in “Eat More” and “Eat Less” Messages.

Of the 106 guidelines analysed in this study, 91 included some level of advice about processing and 105 included nutrient-focused advice. Figure 2 shows the percentage of guidelines that contained at least one occurrence of nutrient-focused messages or processing focused messages in ‘eat more’ (green bars) and ‘eat less’ (red bars) advice. Country-specific data is provided in Supplementary file 4.

 In ‘eat more’ messages, a focus on nutrients was used in two distinct ways: First, ‘positive’ nutrients were mentioned in 69% of all guidelines to promote certain foods or food groups. Within that group, more than half promoted the consumption of ‘positive’ fats (eg, “High-fat fish […] is a good source of polyunsaturated fatty acids, which seem to give protection against coronary heart disease”/Namibia). Other nutrients that were positively promoted included vitamins, calcium and iron (eg, “Consume animal source foods which are source of iron...”/ Bolivia), with a total of 54% of all dietary guidelines conveying such a message. Secondly, 60% encouraged the consumption of foods based on a “low” content of certain ‘negative’ nutrients. Within this group, ‘low-fat’ messages were most common (57% of all guidelines), followed by ‘low-salt’ (29%) and ‘low-sugar’ (28%). Nearly all guidelines (96%) advised to ‘eat less’ of some nutrients, most commonly salt (88%), then fat (82%) and sugar (74%). 63% of guidelines advised to reduce fat consumption in general (eg, “Limit your daily fat intake…”/North Macedonia). When specifying which fat should be eaten less of, 27% emphasised saturated fat, 18% trans-fat, and 18% mentioned other types of fat, for example cholesterol.

An emphasis on food processing was found to be less common than nutrient-centric advice. Out of the 48 guidelines that mentioned the term ‘processed,’ twelve guidelines did so exclusively in combination with ‘meat’ or (rarely) ‘fish’ and not in the context of food processing more general which was only found in 36 guidelines. Implicit references to food processing were found in 42 guidelines. Table 2 shows the range of explicit and implicit terms used to refer to processing in dietary guidelines.

**Table 2:** List of Implicit and Explicit Terms for Processing

**Terms for Processing**	**‘Eat More’ Messages**	**‘Eat Less’ Messages**
Explicit terms	Less processed Minimally processed Unprocessed	Food processing Highly processed Processed Ultra-processed
Implicit terms	Brown [rice/bread] Fresh Home-made In season/seasonal Minimally milled Natural Raw Unbleached Unpolished Unrefined With peel/skin on [fruit and vegetables] Whole [fruits/flour/grain/meal/wheat/wheat flour] Wholemeal/wholegrain	Artificial Canned Commercial Convenience Fast food Frozen Instant Junk foods Packaged Precooked Prepared Ready Ready-cooked Ready-made Refined Tinned Food additives (Chinese salt/flavour enhancer/glucose/monosodium glutamate (MSG)/nitrates and nitrites/preservatives/salt substitutes/sodium bicarbonate/sodium chloride/sodium nitrate/sodium saccharin/Vetsin)

**Figure 3:**
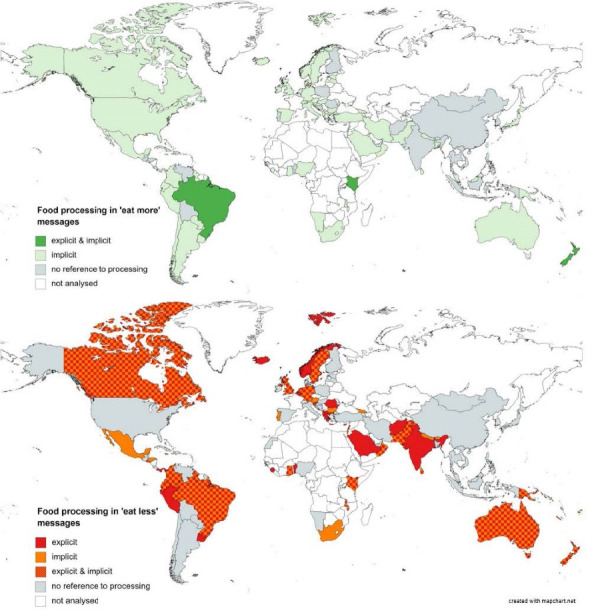
Countries That Refer to Food Processing in “Eat More” and “Eat Less” Messages.

In ‘eat more’ messages, only 5% of guidelines used messages that specifically encouraged the consumption of “minimally processed” or “unprocessed” foods (eg, “Choose […] unprocessed foods”/Brunei Darussalam; “Make […] minimally processed foods the basis of your diet”/Brazil). 75% of all dietary guidelines advised to “eat,” “choose,” “enjoy,” or “prefer” foods with certain characteristics that implied a lack of processing (eg, “Choose fresh, home-made foods….”/Qatar). Many of these messages were restricted to fruit and vegetables (using terms like ‘fresh’ or ‘raw’) or cereal products (using the term ‘whole-grain’ or similar). All six countries that explicitly mentioned minimal processing also used implicit messages (illustrated in dark green in Figure 3).

In ‘eat less’ messages, 45% of all dietary guidelines explicitly mentioned at least one of the specific terms in relation to processing. Out of those, seven countries (Belgium, Brazil, Ecuador, Israel, Maldives, Peru and Uruguay) mentioned “ultra-processing” specifically. Explicit advice regarding food processing was often restricted to particular nutrients, ingredients, or foods (eg, “Limit your intake of processed products high in sodium”/Panama). Overall, 43% of all guidelines conveyed implicit references to processing in their ‘eat less’ messages, including those that mentioned additives or specific characteristics of processed foods (eg, “…avoid: Dried fruits with added […] preservatives”/Fiji). The terms ‘canned,’ ‘refined’ or ‘commercial’ were commonly used to imply processing, as well as the term ‘fast foods’ or ‘packaged’ (eg, “reduce the consumption of packaged products”/Colombia). More than half of the guidelines which explicitly mentioned processing also included implicit messages about products that are usually highly processed (eg, “avoid the consumption of UPFs, fast food and sugar sweetened beverages”/Ecuador). Figure 3 shows the countries that referred to processing explicitly, implicitly or both in their ‘eat more and ‘eat less’ messages.

Based on the phase-1 analysis, two broad patterns could be seen when comparing dietary advice according to country income levels. First, ‘eat more’ messages promoting foods ‘low’ in ‘negative’ nutrients (eg, choose low-fat…) were more common in high-income countries than in low- and middle-income countries^[2]^. While less than half of all low- and middle-income countries used terms like ‘low-fat’ or ‘low-sugar,’ more than three quarters of all high-income countries recommended such foods. Secondly, explicit advice to reduce consumption of processed foods (ie, ‘processed,’ ‘highly processed’ or ‘ultra-processed’) was more common in low- and lower middle-income countries, with 53% of the assessed countries in this income group advising to eat less processed products whereas only 31% of all upper-middle and 51% of all high-income countries used such messages. In high-income countries, this was often limited to advice about processed meat, with only 29% of high-income countries referring to processed foods more generally.

 Looking at publication dates of dietary guidelines, the occurrence of processing-related terms (both implicit and explicit) has been increasing over time. Some guidelines included specific advice to limit processed foods as early as 2004, for example, the dietary guidelines for Guyana from 2004 state “Foods high in salt that should be avoided: Chips, pickled products, processed foods, […] packaged macaroni, […] fast foods & canned foods.” Similarly, Malawi published national dietary guidelines in 2007, advising to “eat locally available or indigenous and fresh foods rather than exotic or highly processed foods.”

###  NOVA Classification of Foods in “Eat Less” Dietary Messages

Most countries (80% of all guidelines) referred to some type of food or beverage in their ‘eat less’ messages. Overall, a large variety of foods and food groups were mentioned in ‘eat less’ messages (see Supplementary file 3). Of these, the most commonly mentioned food group was animal-based foods and products, followed by sweets, sugars and desserts and then beverages. Fiure 4 shows the breakdown of food groups mentioned in ‘eat less’ guidelines according to their NOVA group.

**Figure 4:**
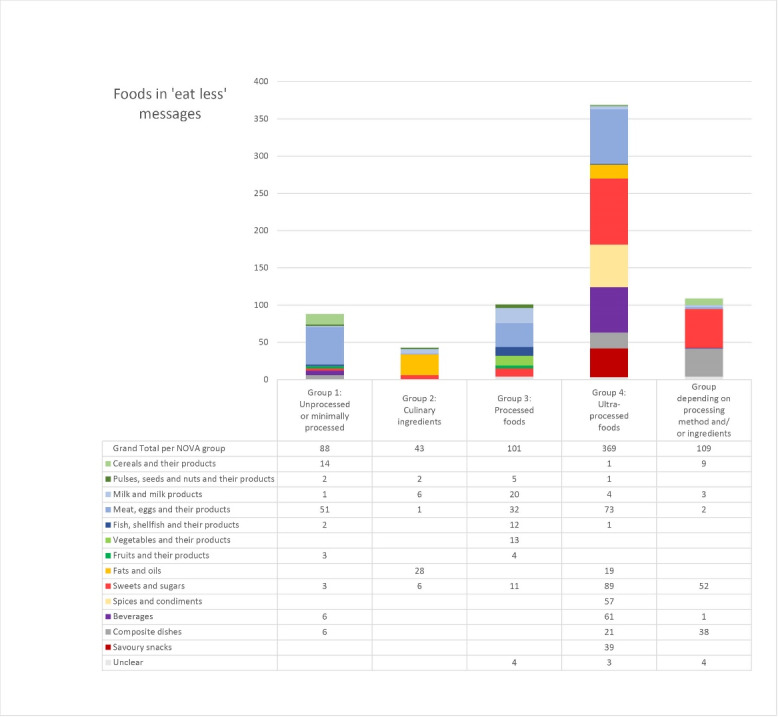
Foods in “Eat Less” Messages by NOVA Classification and Food Group.

Fifty-two percent of all discouraged foods and beverages were UPFs with the most frequently foods mentioned being soft drinks, sweets, sausages, chips/crisps, and margarine. Unprocessed and minimally processed foods were also included in ‘eat less’ messages, as well as processed culinary ingredients. For the latter, butter was the most commonly mentioned item. Regarding unprocessed foods, meat was the most frequent which included references to meat generally or specific types of meat (eg, red meat). For 15% of foods, the classification required further information about ingredients or processing method (eg, freshly baked vs. packaged bread) that was unavailable, and therefore a NOVA-food group could not be assigned (see Supplementary file 3 for NOVA-classification of each term). The most commonly mentioned term in this unclear category was ‘cakes,’ which could be either a handmade dish made mostly with ingredients from group 1, or a manufactured dish, classified as group 3, or 4, depending on the ingredients.

 With respect to the food groups, nearly a third of all discouraged foods were animal-based, which included dairy, eggs, meat, fish, and related products. As an overarching group, animal-sourced foods included the highest proportion of unprocessed foods with more than a fifth falling into this category. These unprocessed foods were almost exclusively meat, either in general or specific types of meat (eg, “Limit red meat consumption”/Greece). In addition, about half of all terms in the ‘Fats and oils’ category were also animal-sourced, namely butter, ghee and lard. The rationale behind these ‘eat less’ messages was sometimes based on the saturated fat content (eg, “Choose and/or prepare foods and drinks: with unsaturated fats (canola, olive, rice bran or vegetable oil, or margarine) instead of saturated fats (butter, cream, lard, dripping, coconut oil)”/New Zealand), but also the fat content generally (eg, “Reduce intake of fats and oils: This includes cooking oil, margarine, butter, meat fat,…”/Jamaica). The second most frequently mentioned term in the ‘Fats and oils’ group was margarine, which is considered ultra-processed.

 Almost one quarter of all terms were classified as ‘sweets and sugars.’ Within this group, more than half of all terms were clearly identifiable as ultra-processed, however, for nearly a third, a NOVA-group could not be assigned based on the available information. For example, terms like ‘cake,’ ‘desserts’ or ‘pastries’ may be home-made, but they may also be store-bought with added emulsifiers or similar and would then qualify as ultra-processed. The ‘beverages’ group consisted mostly of UPFs, namely soft drinks. Less processed drinks included fruit juice or smoothies. Almost a third of foods that belonged to the ‘Composite dish’ category were UPFs (eg, ‘instant noodles’), but for more than half of the terms a NOVA-classification was not possible (eg, ‘burgers’ or ‘fried foods’). When considering plant-based foods, cereal-based products were most commonly mentioned (eg, ‘bread’ or ‘pasta’), but also fruit- and vegetable-derived products (eg, ‘canned vegetables’). Most foods in this group belonged to the third NOVA-group, processed foods. Savoury snacks were almost exclusively considered UPFs and ‘chips/crisps’ was the most commonly discouraged snack.

## Discussion

While nutrient-centred language is widely used in dietary guidelines around the world, information on UPFs was found to be more common than expected. Based on previous research,^7^ it was assumed that only a very small number of guidelines would incorporate such advice. This was confirmed when looking at the term “ultra-processed” alone, which was only used in seven countries. Four of these countries are in South America and may reflect the Brazilian origin of the NOVA framework. This research found that dietary guidelines can incorporate the concept of ultra-processing in three different ways. First, typical characteristics of UPFs (eg, ‘packaged’) can be used to imply that a product is processed (UPF euphemisms). Secondly, specific examples of UPFs (eg, crisps or ice cream) can be named. Thirdly, ‘ultra-processed’ or related terms can be used to explicitly refer to processing levels. Nonetheless, each of these approaches present inconsistencies and limitations in how the concept of ultra-processing is being taken up in dietary guidelines, which we reflect on below.

UPF euphemisms were frequently used in the context of food processing, and they could be interpreted as being more consumer-friendly because such terms may align better with the ideal of ‘food-based’ advice. For example, a term like ‘canned foods’ or ‘packaged snacks’ might be easier to understand than the terms ‘processed’ or ‘ultra-processed’ foods. While these terms refer to clearly visible characteristics (such as packaging) UPF euphemisms may not clearly differentiate between levels of processing. Both canned and packed foods, for instance, could be processed or ultra-processed, thus it is important that clear explanations and examples of ultra-processing are provided. A key point worth explaining to consumers is the nature and purpose of different types of food processing as outlined in the NOVA framework. Specifying euphemistic terms is particularly important in ‘eat more’ messages, as they may otherwise be utilised as yet another marketing opportunity by labelling products using ambiguous (and often unregulated) terms like ‘natural,’ ‘fresh,’ or ‘raw.’ This phenomenon can already be observed with the labelling of highly processed foods as ‘natural’ or ‘wholemeal/grain,’ which tends to be prevalent in products like packaged bread, breakfast cereals or products of the ‘health food’ sector. Due to time and resource constraints, a systematic assessment of UPFs in ‘eat more’ messages was not part of this study, but it appears that the frequent use of such ambiguous terms in dietary guidelines may even promote the consumption of UPFs. For example, Kellogg’s^53^ launched ‘Be Natural®’ as a new brand, marketing ultra-processed cereal bars and flakes as “very high in whole grains” and (central to the brand name) “natural.” Examining the rationale behind the use of implicit or explicit references to processing or whether they are relevant to or understood by consumers was also outside the scope of this project, but the terms identified in this study (Table 2) could inform further analysis of consumer-understanding and suitability of such advice.

One possible explanation for the use of euphemistic terms in dietary guidelines may lie in the ambiguity and imprecision around how different levels of processing are understood and interpreted – both amongst consumers as well as nutritionists and food scientists.^5,6,22^ This ambiguity was also noticeable in our categorisation of foods according to the NOVA framework, where many foods could not be classified as further details were needed about ingredients or their method of preparation. Indeed, depending on the method of preparation, the same food category could be classified at multiple processing levels. Dietary guidelines could address this by including advice about how to differentiate between more artisanal or industrial methods of preparation, for example by recommending to prefer freshly cooked dishes over industrially prepared ready-to-eat meals. This could be aided by including examples of UPFs commonly perceived as healthy (eg, packaged breads and flavoured yoghurts) in addition to the more obvious examples used in many guidelines (eg, crisps and soft drinks).

Similar to the use of euphemistic terms, the persistence of nutrient-level advice could encourage consumption of UPFs. Many guidelines included advice to ‘eat more’ of foods low in salt, sugar and fat. Dietary guidelines (or other nutrient policies) discouraging the consumption of particular nutrients or the consumption of ‘low-nutrient’ products may lead to reformulation of foods to reduce those nutrients.^54^ Changing the composition of UPFs, ie, reformulating them, is a common approach to make products ‘healthier,’ or at least make them appear healthier by using labels like ‘low-fat.’ However, research suggests that products with such claims do not automatically have healthier nutrient profiles.^55,56^ Additionally, such products are often processed in a more substantial way when compared with their full-fat counterparts as additives are used to make up for the lack in taste and texture.^57^ Significant concerns have been raised about the quality of these reformulated foods, such as the use of artificial sweeteners or fat substitutes, and the extent to which reformulated foods are used as a strategy by food companies to deter policies to reduce consumption of UPFs.^57-59^

Considering food-based messages, more than half of the specific foods mentioned in ‘eat less’ advice are UPFs, but group 1 foods such as meat or group 2 products like butter were also discouraged. This raises further questions about the extent to which the concept of ultra-processing is incorporated into dietary advice, and the consistency of its application. In ‘eat less’ advice, three quarters of minimally processed foods and culinary ingredients were animal-based products while only a fifth of UPFs were clearly animal-sourced. Assessing the rationales behind the inclusion of those particular foods was beyond the scope of this project, but possible explanations could be the focus on nutrients (eg, the content of saturated fats) or the incorporation of sustainability issues in dietary guidelines.^45^ Of the seven countries that made specific reference to UPFs or the NOVA framework, only Peru referred to minimally processed foods (“Take care of your weight by consuming rice, pasta […] in moderation”) in its eat less advice. This suggests that use of the NOVA framework can complement the development of food-based dietary guidelines to ensure that the specific foods mentioned align with levels of processing.

Finally, while some dietary guidelines used the specific term “ultra-processed” in their consumer-targeted messages, questions can be raised about the extent to which this concept is clearly communicated to consumers. A key component of consumer-targeted dietary advice is its suitability (ie, whether the advice is clear, achievable, and affordable). Evaluating these aspects was outside the scope of this project, but the results could inform future research. This could include a regional focus as cultural and societal environments differ. Research from South America has shown that while consumers identify most UPFs, some ambiguities and misconceptions remain.^64-66^ For example, milk, which is considered a minimally processed food, was often misclassified as ultra-processed in Argentina, Ecuador and Uruguay.^64,65^ In Brazil, the first country to include explicit advice to avoid UPFs in their dietary guidelines in 2014, a recent study found that consumer behaviour has not changed substantially and identified the need to make dietary advice “more accessible, and actionable to be potentially more effective.”^67^ To ensure that consumers understand messages about UPFs, similar research is needed in other regions, incorporating specific cultural and socio-economic circumstances to ensure that the NOVA framework and concept of ultra-processing is communicated in ways relevant and understandable to communities.

While we can observe a gradual increase in the number of dietary guidelines providing advice about processed foods, our study has not explored the underlying rationales and justifications for a focus on processing. One possible explanation for the persisting focus on nutrients is that nutrient-based research is more likely to inform the creation of dietary guidelines because of the mechanisms behind hierarchical evidence frameworks.^61^ For example, evidence from a randomized controlled trial assessing the effects of a single nutrient would be ranked higher than a cohort study about the consequences of UPF-consumption. And even though research on broader dietary patterns and UPF consumption has been increasing,^60^ a reductive approach centred on nutrients still builds the basis for key measures like recommended daily intakes. A recent study found that even when dietary guidelines provided advice reducing consumption of UPFs, this was usually justified in terms of poor nutrient profiles, as opposed to more upstream explanations of the harms of ultra-processing.^68^ Clearly, nutrient-based research has been an essential field of health science and still plays an important role in the context of diet-related NCDs. Consequently, some types of nutrient-based information for consumers are still useful, for example the advice to reduce intake of simple carbohydrates like sugar. Nevertheless, nutrient-level advice is often decontextualised and might be promoting the consumption of UPFs (eg, reduced-sugar soft drinks, low-fat ready to heat meals) and a more differentiating approach in consumer guidelines appears necessary.

## Conclusion

 Ultra-processing is a concern for population health, though not food processing in general. The NOVA food classification system offers a framework to better understand this difference and became an important instrument in public health research and policy. This study provides the first systematic assessment of how the concept of UPFs is incorporated in dietary advice around the world. This paper documents the diversity of terms and foods associated with this concept, which may provide important insights to the public health nutrition community as to how dietary guidelines for consumers discuss UPFs and the concept of food processing, and the issues and ambiguities with current approaches.

 Due to time and resource constraints, an analysis of the full text of detailed guidelines was not possible, and it is acknowledged that some countries may discuss UPFs in more detail than what is found in the consumer-targeted messages (eg, Brazil and Canada) or that some may have updated their guidelines. Additionally, visual food guides and non-English or non-German documents were excluded. Consequently, some guides may not be represented in a way that corresponds with how they communicate in visual or native language materials. Future research could focus on such elements of consumer-directed dietary advice and native speakers or translators could illuminate the representation of UPFs in dietary advice in regions that were not well captured by this project (eg, South-Eastern and Eastern Asia).

Coinciding with the growing evidence base around the harms of UPFs, this study found that the incorporation of processing-based messages in consumer-directed advice has been increasing over the last decade. Nevertheless, in the context of consumer-targeted dietary guidelines, this research has shown that there are inconsistencies in how the concept of ultra-processing is incorporated into dietary advice. The results suggest that while specific examples of UPFs are commonly used in consumer-dietary guidelines, this is not matched with a similar focus on or explanation of processing levels. An important area for future research will be the development of dietary guidelines that communicate the concept of ultra-processing in consumer-friendly formats. Dietary guidelines are an important instrument in nutrition policy, but to achieve equitable, population-level improvements in nutrition, policy makers must address food environment levers as well.^69^ It is therefore important that messages about UPFs are matched with supportive policies to ensure that the advice is not only clear, but also achievable, both from a financial and a practical standpoint.

## Acknowledgements

 PM receives income through an Alfred Deakin Postdoctoral Research Fellowship provided by Deakin University.

## Ethical issues

 The material analysed for this research is publicly available and contains no personal information about any individuals. Formal ethics approval was therefore not required.

## Competing interests

 Authors declare that they have no competing interests.

## Authors’ contributions

 DK and JLN developed the project aims and research design. DK collected and analysed the data, with input from JLN and PM. DK wrote the first draft of the paper, and all authors collaborated on subsequent iterations. All authors read and approved the final manuscript.

## Endnotes

 [1] The first author’s first language. [2] The analysis by income levels excluded countries that are part of the ‘Pacific Community’ because the income levels of these countries vary from lower middle to high income. Fiji officially refers to their own guide, therefore the Fijian guidelines were included.

## Supplementary files


Supplementary file 1. Dietary Guideline Sources.

Supplementary file 2. Coding Framework.

Supplementary file 3. NOVA and FAO Food Group Classifications for Food Terms.

Supplementary file 4. Country-Level Data Summary.
